# Development and internal validation of a Cox-based recurrence risk prediction model after endovenous laser treatment for lower-extremity varicose veins

**DOI:** 10.3389/fsurg.2026.1935570

**Published:** 2026-10-07

**Authors:** Mingxu Ou, Jiang Xu, Kelong Shi, Xiping Zhao, Xianlu Cui, Danqing Wang

**Affiliations:** 1 Department of Cardiac and Great Vascular Surgery, Tongling People’s Hospital, Tongling, Anhui, China; 2 Bengbu Medical University, Bengbu, Anhui, China

**Keywords:** Cox regression, endovenous laser treatment, internal validation, prediction model, recurrence, varicose veins

## Abstract

**Background:**

Recurrence after endovenous laser treatment (EVLT) remains clinically important. We therefore developed and internally validated a Cox-based model for medium- to long-term recurrence after EVLT.

**Methods:**

This single-center retrospective cohort included 183 patients treated between March 2017 and December 2023; 78 experienced recurrence and 105 did not. The primary Cox proportional hazards model retained age, sex, body mass index (BMI), disease duration, family history, perforator vein incompetence, preoperative great saphenous vein (GSV) diameter, and Venous Clinical Severity Score (VCSS). Perforator vein incompetence was coded as Yes = present and No = absent, with No as the reference category. A nomogram estimated 12, 24, and 36 month recurrence risk. Model performance was evaluated using ROC analysis, calibration plots, bootstrap internal validation, and decision curve analysis (DCA).

**Results:**

In the multivariable Cox model, BMI (HR 1.122, 95% CI 1.013–1.242; *P* = 0.027), disease duration (HR 1.024, 95% CI 1.000–1.048; *P* = 0.046), and family history (HR 1.635, 95% CI 1.017–2.628; *P* = 0.042) were associated with higher recurrence hazard, whereas perforator vein incompetence (Yes = present vs. No = absent) was inversely associated with recurrence hazard (HR 0.351, 95% CI 0.193–0.639; *P* < 0.001). Age did not reach conventional significance after adjustment (HR 1.024, 95% CI 0.999–1.050; *P* = 0.064). The model ROC AUC was 0.799 (95% CI 0.734–0.864). The original and bootstrap-corrected C-indices were 0.727 and 0.694, respectively. Detailed bootstrap validation yielded a corrected calibration slope of 0.807, indicating some optimism. The supplied 36 month DCA showed the model curve overlapping the treat-none strategy across the displayed threshold range, so incremental net benefit was not demonstrated in this analysis.

**Conclusions:**

The revised Cox-based model provides moderate discrimination and generally reasonable calibration after internal validation, but its performance attenuates after optimism correction and clinical net benefit was not demonstrated by the supplied 36 month DCA. External validation is required before clinical implementation, and the inverse association observed for perforator vein incompetence should be interpreted cautiously and confirmed in independent cohorts.

## 1 Introduction

Lower-extremity varicose veins are a common manifestation of chronic venous disease and arise from sustained venous hypertension and reflux. Reported prevalence varies substantially by population, age, sex, and diagnostic criteria (1, 2). Endovenous laser treatment (EVLT) is widely used for great saphenous vein disease because it achieves target-vein closure with limited surgical trauma (3, 4). Nevertheless, recurrent varicosities remain an important medium- to long-term problem after intervention, and recurrence reflects both patient-related and anatomic/hemodynamic factors (5, 6).

Previous studies have linked recurrence or post-ablation failure to factors such as age, obesity, vein diameter, disease severity, and treatment characteristics (5, 7, 10–12). However, identifying individual associations is different from constructing a prediction model that integrates multiple predictors, preserves time-to-event information, and demonstrates discrimination, calibration, internal validity, and clinical utility. To preserve time-to-event information and reduce reliance on data-derived cutoffs, the present study uses a Cox proportional hazards model as the sole primary modeling framework. Continuous predictors are retained on their original scales, the nomogram incorporates all predictors in the primary multivariable model rather than selectively retaining only statistically significant variables, and model performance is assessed with ROC discrimination, calibration at 12, 24, and 36 months, bootstrap internal validation, and DCA. Additional sensitivity, collinearity, correlation, and nonlinear-association analyses are provided in the Supplementary Material.

## 2 Materials and methods

### 2.1 Study design and patient population

Patients with primary lower-extremity varicose veins who underwent great saphenous vein EVLT at Tongling People's Hospital between March 2017 and December 2023 were identified through postoperative telephone follow-up and hospital-based reassessment. The analytical cohort comprised 183 patients, including 78 with recurrence and 105 without recurrence according to the Recurrent Varicose Veins After Surgery (REVAS) criteria.

Inclusion criteria were: (1) primary varicose veins attributed to venous valvular dysfunction; (2) treatment including EVLT; (3) at least one year between EVLT and follow-up; (4) ability and willingness to complete the follow-up questionnaire and duplex ultrasound examination; and (5) complete clinical data for the revised analysis. Exclusion criteria were secondary varicose veins related to post-thrombotic syndrome, severe iliac vein compression, or Klippel-Trenaunay syndrome; treatment without EVLT; follow-up shorter than one year; inability to complete follow-up; or unavailable clinical data.

### 2.2 Ultrasonography, recurrence definition, and outcome time

Duplex ultrasound was performed with the SonoScape S15 system. Examination assessed the saphenofemoral junction, great saphenous vein trunk and tributaries, anterior accessory saphenous vein, calf perforators, and clinically visible varicosities. GSV diameter was measured, and reflux was assessed with provocative maneuvers. Follow-up ultrasound measurements were performed by a single sonographer.

Recurrence was defined according to REVAS criteria and included newly developed, residual, or recurrent varicose veins after prior treatment. For time-to-event modeling, postoperative follow-up time was measured from EVLT to recurrence detection in recurrent patients and from EVLT to the last documented follow-up in patients without recurrence; non-recurrent patients were censored at their last follow-up.

### 2.3 Candidate predictors and data quality

The revised dataset included age, sex, BMI, disease duration, family history of varicose veins, perforator vein incompetence, preoperative GSV diameter, VCSS, compression-stocking duration and daily wear time, affected side, lifestyle status, surgical approach, hypertension, chronic cardiac dysfunction, alcohol consumption, smoking, prior lower-extremity or pelvic surgery, regular exercise, and a clinical-class variable coded from 3 to 6. The primary prediction model included age, sex, BMI, disease duration, family history, perforator status, GSV diameter, and VCSS. The clinical-class variable was evaluated in sensitivity analysis because the highest categories were sparsely represented. The coding of perforator vein incompetence was rechecked against the original analytical dataset and its variable definition. In the source dataset, 0 denoted the presence of perforator vein incompetence and 1 denoted its absence. For clinical interpretability, the variable was relabeled as Yes = present and No = absent, with No used as the reference category in the Cox analyses.

There were no missing values in the revised analytical dataset (Supplementary Table S1). Outlier screening identified a small number of flagged values in BMI, disease duration, VCSS, compression-stocking duration, and daily stocking-wear time (Supplementary Table S5).

### 2.4 Statistical analysis and model development

Revised analyses were performed in R (version 4.5.3). Continuous variables are summarized as mean ± standard deviation when approximately symmetric or as median (interquartile range) when skewed; categorical variables are summarized as percentages. Baseline group comparisons are reported using the *P* values from the revised analysis. Univariable Cox proportional hazards models were used to describe associations with recurrence-free survival. The primary multivariable Cox model contained eight clinically relevant predictors: age, sex, BMI, disease duration, family history, perforator status, GSV diameter, and VCSS.

The Cox model was selected as the primary framework because recurrence timing varied across patients and censoring was present. Logistic regression and ROC-derived dichotomization were not used for model derivation in the revised analysis. Continuous variables were retained on their original scales, and the model coefficients were directly translated into a nomogram estimating 12, 24, and 36 month recurrence risk. All eight primary-model predictors were retained in the nomogram to avoid *post hoc* selection based solely on *P* values.

No formal *a priori* model-development sample-size calculation was available in the supplied materials. The revised cohort contained 78 recurrence events for eight primary predictors (approximately 9.75 events per predictor), and bootstrap internal validation was used to quantify optimism. Discrimination was summarized using ROC AUC and Harrell-type concordance estimates from the supplied validation output; calibration was examined at 12, 24, and 36 months. DCA evaluated 36 month clinical net benefit. Multicollinearity was examined with variance inflation factors (VIFs), pairwise correlations were inspected, and exploratory restricted cubic spline plots assessed the shape of associations for BMI and disease duration. Sensitivity analyses excluded time-zero observations and additionally adjusted for the clinical-class variable. Two-sided *P* < 0.05 was considered statistically significant.

### 2.5 Treatment and quality control

All procedures were performed by senior attending physicians. The standard approach combined EVLT with high ligation of the great saphenous vein, with adjunctive stab phlebectomy for severe varicose clusters. Postoperative compression bandaging was used for three days, followed by medical compression stockings. Patients were advised to continue daytime stocking use for at least six months. A single sonographer performed follow-up ultrasound measurements to improve measurement consistency.

## 3 Results

### 3.1 Cohort characteristics

Among 183 patients, 78 (42.6%) had recurrent varicose veins and 105 (57.4%) did not. Patients with recurrence were older (64.28 ± 9.36 vs. 56.49 ± 9.50 years; *P* < 0.001), had higher BMI (median 24.03 vs. 23.39 kg/m^2^; *P* = 0.042), and had longer disease duration (median 10 [IQR 8–20] vs. 7 [IQR 5–10] years; *P* < 0.001). Family-history distribution differed between groups (*P* = 0.034), as did perforator status (*P* < 0.001), compression-stocking duration (*P* = 0.033), and clinical class (*P* = 0.010). GSV diameter (*P* = 0.918) and VCSS (*P* = 0.204) did not differ significantly at baseline (Table 1).

**Table 1:** Baseline characteristics according to recurrence status.

Variable	Overall (*n* = 183)	Non-recurrence (*n* = 105)	Recurrence (*n* = 78)	*P* value
Age, years	59.81 ± 10.18	56.49 ± 9.5	64.28 ± 9.36	<0.001
BMI, kg/m^2^	23.53 (22.23–25.1)	23.39 (22.15–24.73)	24.03 (22.48–25.44)	0.042
Disease duration, years	10 (5–10)	7 (5–10)	10 (8–20)	<0.001
GSV diameter, mm	10.53 (8.91–12.62)	10.26 (8.93–12.87)	11.01 (8.82–12.56)	0.918
VCSS, points	8 (5–12)	8 (5–12)	9 (6–12)	0.204
Compression stocking use, months	6 (3–12)	6 (6–12)	6 (3–12)	0.033
Daily compression stocking wear, h/day	12 (12–12)	12 (12–12)	12 (12–12)	0.172
Sex	Female (46.4%); Male (53.6%)	Female (51.4%); Male (48.6%)	Female (39.7%); Male (60.3%)	0.156
Family history of varicose veins	No (64.5%); Yes (35.5%)	No (71.4%); Yes (28.6%)	No (55.1%); Yes (44.9%)	0.034
Perforator vein incompetence	No (9.3%); Yes (90.7%)	No (0%); Yes (100%)	No (21.8%); Yes (78.2%)	<0.001
Affected side	Left (50.8%); Right (49.2%)	Left (56.2%); Right (43.8%)	Left (43.6%); Right (56.4%)	0.124
Unhealthy lifestyle	No (63.4%); Yes (36.6%)	No (68.6%); Yes (31.4%)	No (56.4%); Yes (43.6%)	0.125
Surgical approach	EVLT + high ligation (69.4%); EVLT + high ligation + phlebectomy (30.6%)	EVLT + high ligation (68.6%); EVLT + high ligation + phlebectomy (31.4%)	EVLT + high ligation (70.5%); EVLT + high ligation + phlebectomy (29.5%)	0.905
Hypertension	No (69.9%); Yes (30.1%)	No (75.2%); Yes (24.8%)	No (62.8%); Yes (37.2%)	0.099
Chronic cardiac dysfunction	No (92.9%); Yes (7.1%)	No (94.3%); Yes (5.7%)	No (91%); Yes (9%)	0.577
Alcohol consumption	No (74.9%); Yes (25.1%)	No (80%); Yes (20%)	No (67.9%); Yes (32.1%)	0.092
Smoking	No (72.7%); Yes (27.3%)	No (76.2%); Yes (23.8%)	No (67.9%); Yes (32.1%)	0.285
Prior lower-extremity or pelvic surgery	No (75.4%); Yes (24.6%)	No (77.1%); Yes (22.9%)	No (73.1%); Yes (26.9%)	0.647
Regular exercise	No (59.6%); Yes (40.4%)	No (59%); Yes (41%)	No (60.3%); Yes (39.7%)	0.990
Clinical class (levels 3–6)	3 (6.6%); 4 (89.6%); 5 (2.7%); 6 (1.1%)	3 (7.6%); 4 (92.4%); 5 (0%); 6 (0%)	3 (5.1%); 4 (85.9%); 5 (6.4%); 6 (2.6%)	0.010

Values are shown as mean ± SD for age, median (IQR) for other continuous variables, and category percentages for categorical variables, as supplied in the revised analysis. BMI, body mass index; GSV, great saphenous vein; VCSS, Venous Clinical Severity Score. Perforator vein incompetence was coded as Yes = present and No = absent.

### 3.2 Univariable Cox analysis

In univariable Cox analysis, age (HR 1.046 per year, 95% CI 1.024–1.069; *P* < 0.001), BMI (HR 1.114 per kg/m^2^, 95% CI 1.015–1.222; *P* = 0.023), disease duration (HR 1.044 per year, 95% CI 1.023–1.066; *P* < 0.001), family history (HR 1.622, 95% CI 1.037–2.535; *P* = 0.034), and alcohol consumption (HR 1.693, 95% CI 1.051–2.726; *P* = 0.030) were associated with higher recurrence hazard. Perforator vein incompetence (Yes = present vs. No = absent) was inversely associated with recurrence hazard (HR 0.231, 95% CI 0.134–0.397; *P* < 0.001). The linear contrast of clinical class was significant, whereas GSV diameter and VCSS were not significant in univariable analysis (Table 2). VCSS was not included in the clinical-class-adjusted sensitivity model because it was not supplied in the analysis file.

**Table 2:** Univariable Cox proportional hazards analysis of recurrence.

Variable	HR	95% CI	*P* value
Age (per year)	1.046	1.024–1.069	<0.001
Male (vs. Female)	1.314	0.832–2.074	0.241
BMI (per kg/m^2^)	1.114	1.015–1.222	0.023
Disease duration (per year)	1.044	1.023–1.066	<0.001
Family history (Yes vs. No)	1.622	1.037–2.535	0.034
Perforator vein incompetence (Yes vs. No)	0.231	0.134–0.397	<0.001
GSV diameter (per mm)	1.027	0.932–1.131	0.591
VCSS (per point)	1.049	0.990–1.112	0.104
Clinical class (linear contrast)	7.709	2.369–25.091	<0.001
Clinical class (quadratic contrast)	0.729	0.278–1.913	0.521
Clinical class (cubic contrast)	0.527	0.257–1.082	0.081
Right side (vs. Left)	1.472	0.940–2.304	0.091
Unhealthy lifestyle (Yes vs. No)	1.342	0.855–2.105	0.201
EVLT + high ligation + phlebectomy (vs. EVLT + high ligation)	0.991	0.608–1.613	0.970
Hypertension (Yes vs. No)	1.236	0.780–1.959	0.367
Chronic cardiac dysfunction (Yes vs. No)	0.985	0.452–2.150	0.971
Alcohol consumption (Yes vs. No)	1.693	1.051–2.726	0.030
Smoking (Yes vs. No)	1.106	0.686–1.782	0.680
Compression stocking use (per month)	0.989	0.967–1.012	0.336
Daily stocking wear (per hour)	0.938	0.866–1.016	0.118
Prior lower-extremity/pelvic surgery (Yes vs. No)	1.193	0.723–1.968	0.490
Regular exercise (Yes vs. No)	0.980	0.623–1.543	0.932

HR > 1 indicates higher recurrence hazard; HR < 1 indicates lower recurrence hazard relative to the stated reference category. The clinical-class variable was analyzed with polynomial contrasts in the supplied univariable output.

### 3.3 Multivariable Cox model and nomogram

The primary multivariable Cox model included all eight prespecified predictors. BMI (HR 1.122, 95% CI 1.013–1.242; *P* = 0.027), disease duration (HR 1.024, 95% CI 1.000–1.048; *P* = 0.046), and family history (HR 1.635, 95% CI 1.017–2.628; *P* = 0.042) remained associated with higher recurrence hazard. Perforator vein incompetence (Yes = present vs. No = absent) remained inversely associated with recurrence hazard (HR 0.351, 95% CI 0.193–0.639; *P* < 0.001). Age was associated with recurrence in univariable analysis but did not reach conventional statistical significance after adjustment (HR 1.024, 95% CI 0.999–1.050; *P* = 0.064). Sex, GSV diameter, and VCSS were also not independently significant (Table 3; Supplementary Figure S2).

**Table 3:** Multivariable Cox proportional hazards model used for prediction.

Variable	HR	95% CI	*P* value
Age (per year)	1.024	0.999–1.050	0.064
Male (vs. Female)	0.936	0.576–1.521	0.788
BMI (per kg/m^2^)	1.122	1.013–1.242	0.027
Disease duration (per year)	1.024	1.000–1.048	0.046
Family history (Yes vs. No)	1.635	1.017–2.628	0.042
Perforator vein incompetence (Yes vs. No)	0.351	0.193–0.639	<0.001
GSV diameter (per mm)	1.022	0.926–1.128	0.666
VCSS (per point)	1.040	0.977–1.107	0.223

Reference categories were Female for sex, No for family history, and No for perforator vein incompetence. Perforator vein incompetence was coded as Yes = present and No = absent. Continuous predictors were modeled per one-unit increase.

All eight variables in the primary Cox model were incorporated into the nomogram, which provides estimated 12, 24, and 36 month recurrence probabilities from the total-point score (Figure 1). This coefficient-based approach avoids the previous use of ROC-derived cutoffs for individual predictors.

**Figure 1:**
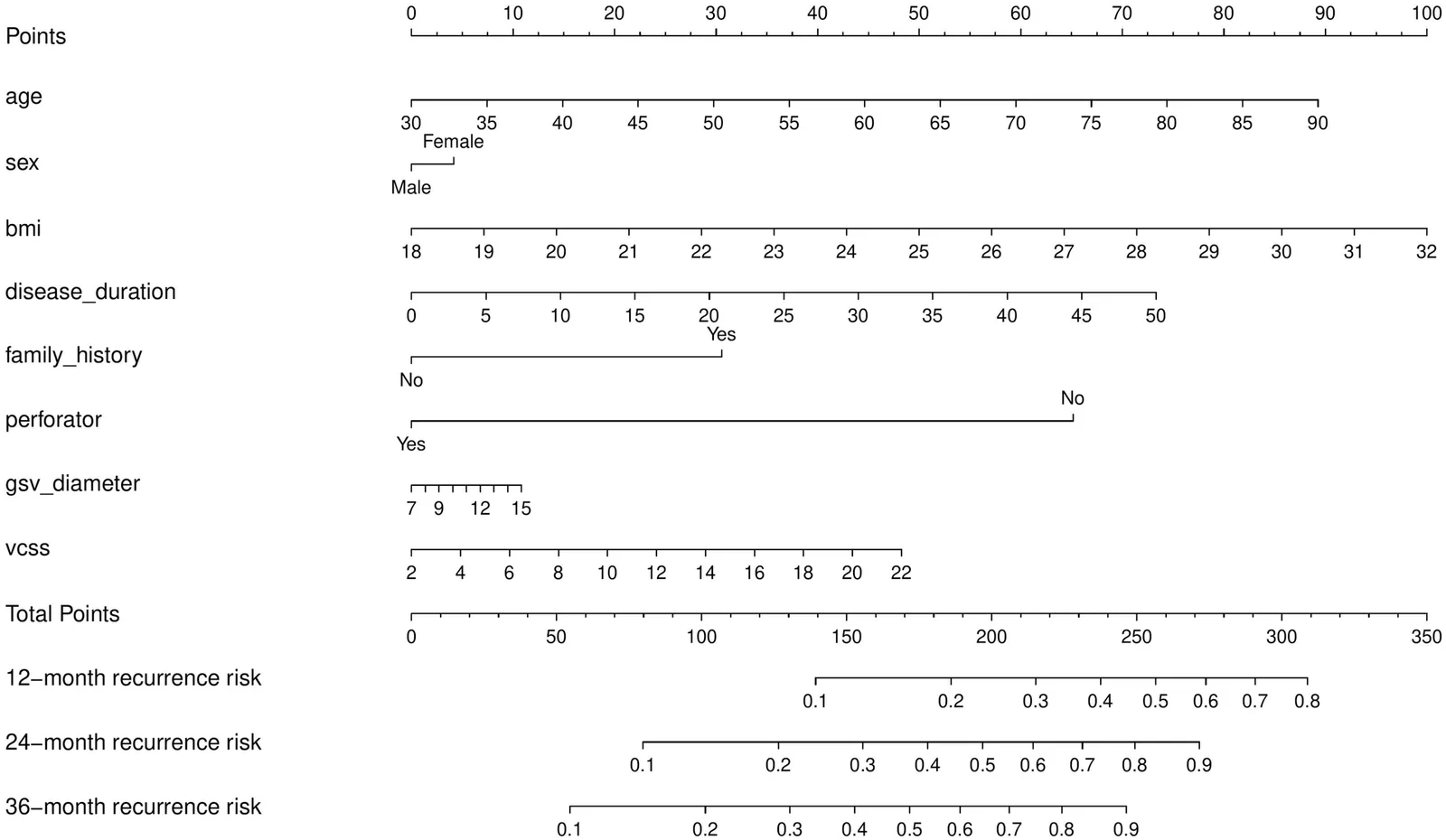
Cox-model nomogram for estimated 12, 24, and 36 month recurrence risk after EVLT. Predictors include age, sex, BMI, disease duration, family history, perforator vein incompetence, GSV diameter, and VCSS. In the nomogram, “perforator” denotes perforator vein incompetence, coded as Yes = present and No = absent.

### 3.4 Discrimination, calibration, and clinical utility

The model ROC curve yielded an AUC of 0.799 (95% CI 0.734–0.864), indicating moderate discrimination (Figure 2). The separate validation summary reported an original C-index of 0.7267 and a bootstrap-corrected C-index of 0.6935 (Supplementary Table S6).

**Figure 2:**
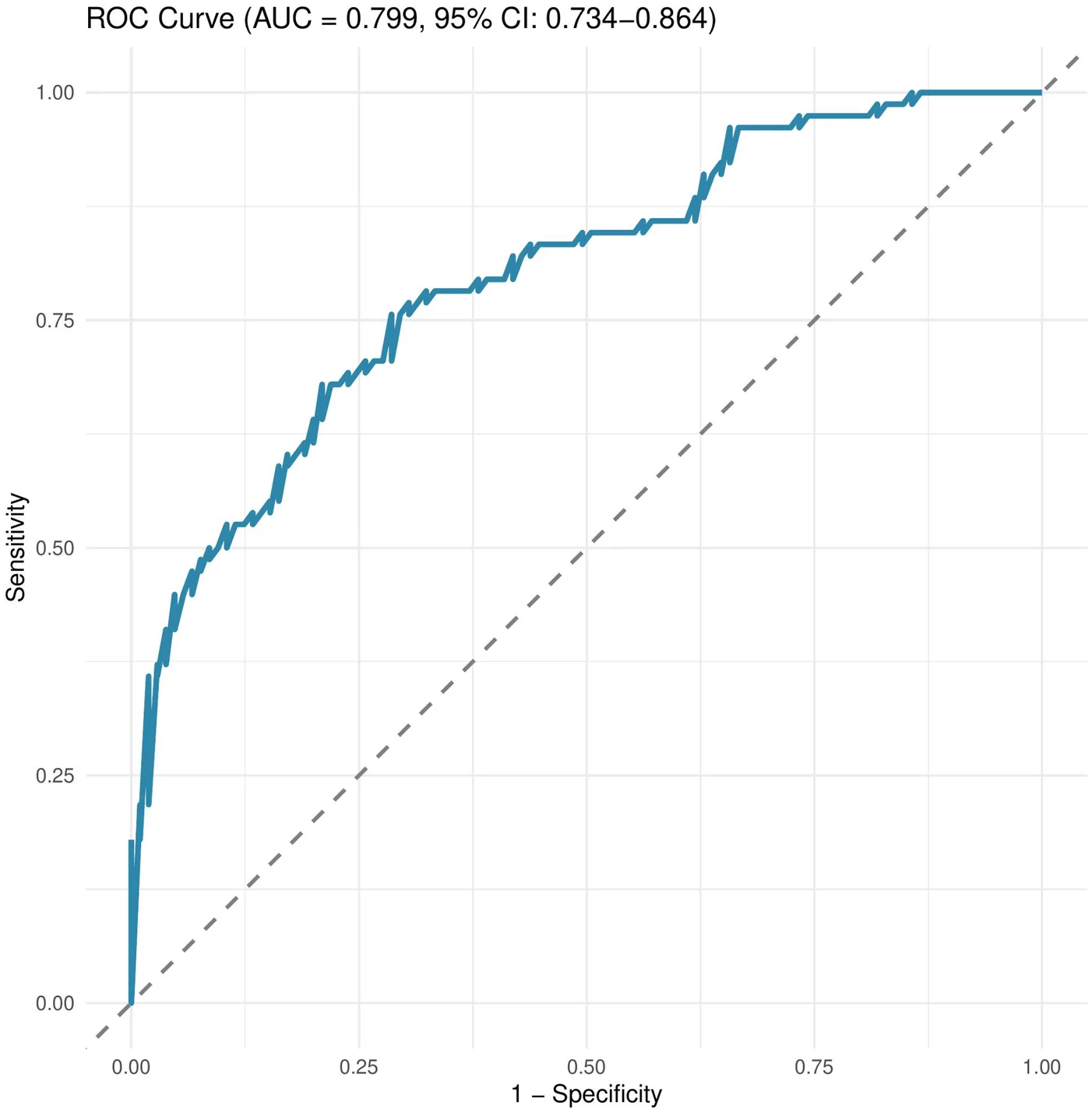
Receiver operating characteristic curve for the revised prediction model (AUC 0.799, 95% CI 0.734−0.864).

Calibration plots on the recurrence-free survival scale showed overall agreement between predicted and observed 12, 24, and 36 month outcomes, with visible deviation at some higher predicted probabilities (Figure 3). In detailed bootstrap validation, the apparent calibration slope was 1.000 and the optimism-corrected slope was 0.807 (Supplementary Table S2). The compact validation summary reported a slope of 0.821 (Supplementary Table S6); Both estimates indicate some overfitting/optimism after internal validation.

**Figure 3:**
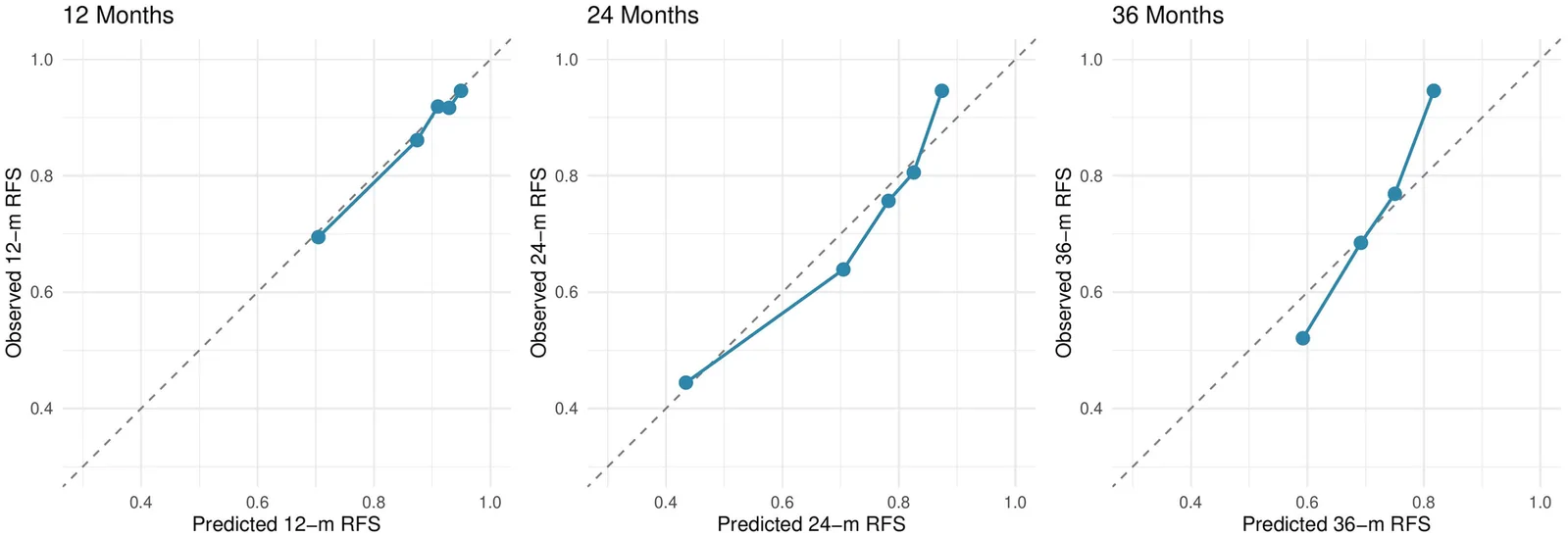
Calibration plots comparing predicted and observed recurrence-free survival (RFS) at 12, 24, and 36 months.

In the supplied 36 month DCA, the model net-benefit curve (risk36) overlapped the treat-none strategy across the displayed threshold probabilities and did not exceed the treat-all strategy where the latter was positive (Figure 4). Accordingly, this analysis did not demonstrate incremental clinical net benefit for the model at 36 months.

**Figure 4:**
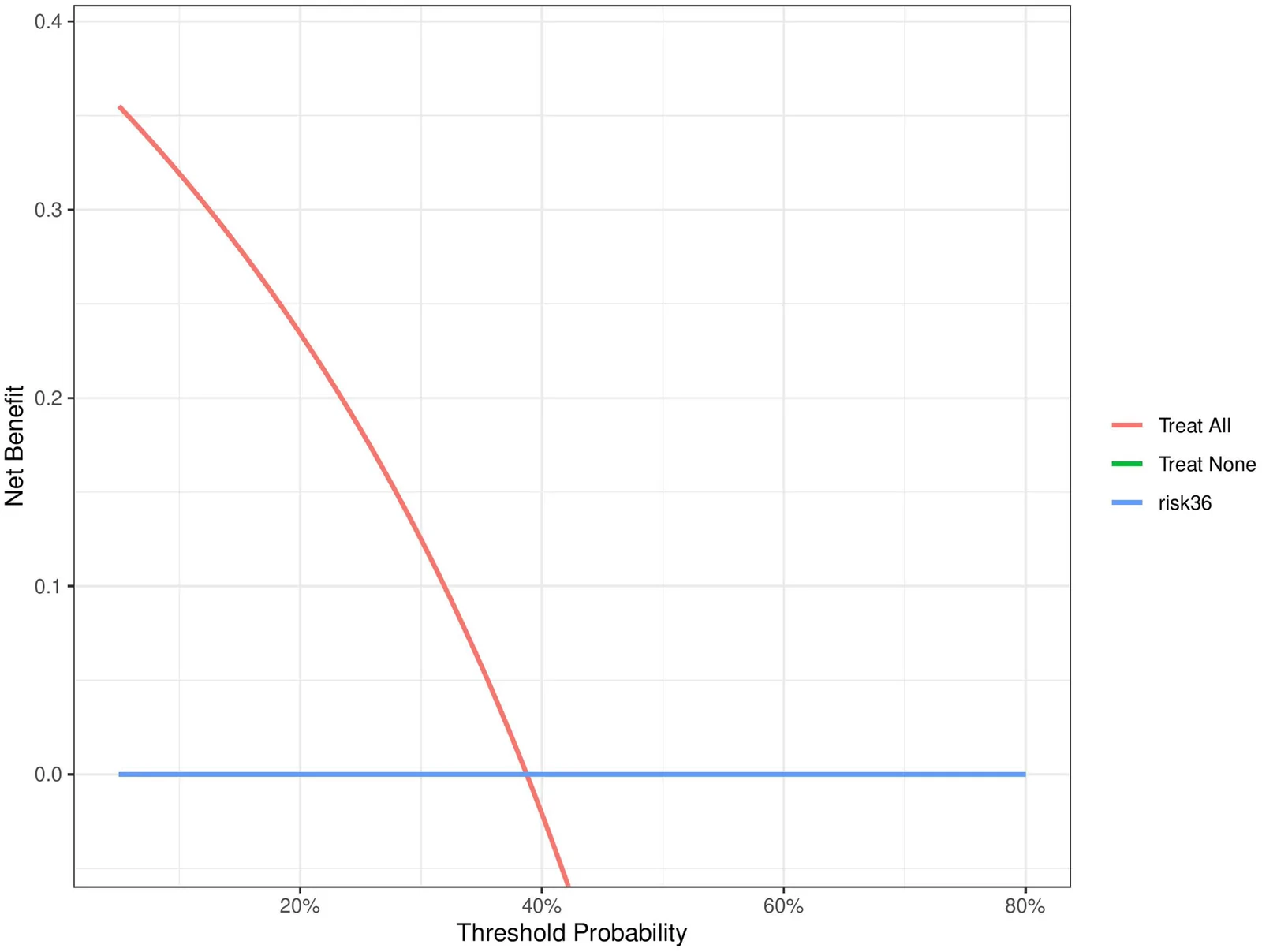
Decision curve analysis for 36 month predicted risk. The supplied model curve (risk36) overlaps the treat-none line across the displayed threshold range.

### 3.5 Model diagnostics, sensitivity analyses, and supplementary analyses

VIFs were low for all primary predictors (maximum 1.076), providing no evidence of problematic multicollinearity (Supplementary Table S4). Pairwise correlations among age, BMI, disease duration, GSV diameter, and VCSS were generally small; the largest displayed correlation was between age and disease duration (r = 0.52) (Supplementary Figure S5). This moderate correlation may partly explain why age was strongly associated with recurrence in univariable analysis but attenuated after multivariable adjustment.

Sensitivity analyses were broadly directionally consistent but showed attenuation for some predictors (Supplementary Table S3). After excluding time-zero observations, disease duration (*P* = 0.032), family history (*P* = 0.033), and perforator status (*P* < 0.001) remained significant, whereas BMI became borderline (*P* = 0.056). In the model additionally adjusted for clinical class, BMI (*P* = 0.013), disease duration (*P* = 0.036), and perforator status (*P* < 0.001) remained significant, whereas family history was attenuated (*P* = 0.067).

Kaplan–Meier curves showed different recurrence-free survival by family history (log-rank *P* = 0.033) and by the supplied perforator-status coding (*P* < 0.0001) (Supplementary Figure S1). Exploratory restricted cubic spline plots suggested an increasing association of BMI with recurrence hazard and an increase in hazard with disease duration that appeared to flatten at longer durations; confidence intervals were wide at the extremes, and no formal nonlinearity *P* value was supplied (Supplementary Figure S3). In the Wald-based importance analysis, perforator status contributed 39.27% of relative importance, followed by BMI (16.32%), family history (13.77%), disease duration (13.33%), age (11.48%), VCSS (4.96%), GSV diameter (0.62%), and sex (0.24%) (Supplementary Table S7; Supplementary Figure S4).

## 4 Discussion

This study developed a single time-to-event prediction framework for recurrence after EVLT. The primary Cox model retained continuous predictors, directly used multivariable coefficients in the nomogram, included all eight prespecified predictors, and was evaluated using discrimination, calibration, bootstrap internal validation, and DCA.

BMI, disease duration, and family history were independently associated with higher recurrence hazard. These findings are biologically and clinically plausible and are broadly consistent with prior work on aging, obesity, venous hemodynamics, and familial susceptibility (7–15). Hemodynamic approaches to chronic venous disease and mechanistic evidence regarding venous hypertension, altered flow, and venous wall remodeling further highlight the relevance of venous hemodynamics (16, 17). However, age itself was no longer statistically significant after adjustment, and GSV diameter and VCSS were not independent predictors in the primary model. Therefore, the results should not be interpreted as confirming every previously proposed risk factor in this cohort.

The coding of perforator vein incompetence was rechecked against the original analytical dataset and its variable definition. The source variable was coded as 0 = presence and 1 = absence of perforator vein incompetence; for analysis and presentation, it was relabeled as Yes = present and No = absent, with No as the reference category. Thus, the inverse association observed in both univariable and multivariable Cox analyses is consistent with the verified dataset coding rather than resulting from inversion of the reference categories. Nevertheless, the direction of this association is clinically counterintuitive and should be interpreted cautiously. In particular, it should not be interpreted as evidence that perforator vein incompetence has a causal protective effect against recurrence. The marked imbalance in this variable (166 patients with and 17 without perforator vein incompetence), together with the fact that all 17 patients without perforator vein incompetence experienced recurrence, may have contributed to the magnitude and direction of the observed association. Confirmation in independent cohorts is therefore required.

The model showed an ROC AUC of 0.799, but optimism correction reduced the C-index from 0.727 to 0.694 and the corrected calibration slope was approximately 0.81. These findings support moderate, not high, predictive performance. The calibration plots were reasonably aligned overall, but deviations at higher predicted probabilities and the difference between apparent and corrected metrics suggest overfitting. The DCA is especially important: in the supplied 36 month analysis, the model did not provide additional net benefit over treat-none across the displayed threshold range. Accordingly, the nomogram should be regarded as an internally evaluated research model rather than a clinically validated decision tool.

Several supplementary analyses support model stability while also illustrating uncertainty. VIFs were close to 1, and most pairwise correlations were weak, although age and disease duration were moderately correlated. Sensitivity analyses preserved the direction of the main coefficients, but BMI and family history crossed the conventional significance threshold under specific alternative specifications. The spline plots also suggest that simple linear effects may not fully capture disease-duration behavior at longer durations. These findings argue for cautious interpretation rather than threshold-based clinical recommendations.

This study has limitations. It is a single-center retrospective cohort with 183 patients and 78 recurrence events, and no external validation cohort was available. Some clinically relevant design information, including complete screening-flow counts and blinding/adjudication details, was not available in the retrospective study records. Although the dataset definition and coding of perforator vein incompetence were verified, the marked imbalance of this variable and its clinically counterintuitive inverse association warrant cautious interpretation and independent confirmation. The primary model was internally validated only, and the absence of demonstrated DCA net benefit limits claims of clinical usefulness. Future work should externally validate the model, confirm predictor definitions and coding, assess calibration across independent populations, and evaluate whether model-guided management improves patient-important outcomes.

## 5 Conclusions

A Cox-based model incorporating age, sex, BMI, disease duration, family history, perforator vein incompetence, GSV diameter, and VCSS was developed to estimate 12, 24, and 36 month recurrence risk after EVLT. The model showed moderate discrimination and reasonable but imperfect internal calibration; bootstrap correction demonstrated measurable optimism, and the supplied 36-month DCA did not show incremental net benefit. The model should therefore be considered preliminary and requires external validation before clinical use. Although the coding of perforator vein incompetence was verified in the original analytical dataset, its inverse association with recurrence should be interpreted cautiously and confirmed in independent cohorts.

## Data Availability

The raw data supporting the conclusions of this article will be made available by the authors, without undue reservation.
