# Supplementary material for: Development and internal validation of a Cox-based recurrence risk prediction model after endovenous laser treatment for lower-extremity varicose veins
Source: Front Surg. 2026 Oct 7;13:1935570. doi: 10.3389/fsurg.2026.1935570 (PMC13683088; doi:10.3389/fsurg.2026.1935570)
Supplement: Supplementary file 1 [file Supplementaryfile1.docx]

**Supplementary Material**

**Development and internal validation of a Cox-based recurrence risk prediction model after endovenous laser treatment for lower-extremity varicose veins**

*All supplementary tables and figures are cited in the revised main manuscript.*

**Supplementary Tables**

**Table S1. Missing-data summary for the revised analytical dataset**

| **Variable** | **Missing n** | **Missing (%)** |
| --- | --- | --- |
| Recurrence | 0 | 0.0% |
| Follow Time | 0 | 0.0% |
| Sex | 0 | 0.0% |
| Age, years | 0 | 0.0% |
| BMI, kg/m² | 0 | 0.0% |
| Disease duration, years | 0 | 0.0% |
| Family history of varicose veins | 0 | 0.0% |
| Perforator status | 0 | 0.0% |
| GSV diameter, mm | 0 | 0.0% |
| VCSS, points | 0 | 0.0% |
| Clinical class (levels 3-6) | 0 | 0.0% |
| Affected side | 0 | 0.0% |
| Unhealthy lifestyle | 0 | 0.0% |
| Surgical approach | 0 | 0.0% |
| Hypertension | 0 | 0.0% |
| Chronic cardiac dysfunction | 0 | 0.0% |
| Alcohol consumption | 0 | 0.0% |
| Smoking | 0 | 0.0% |
| Compression stocking use, months | 0 | 0.0% |
| Daily compression stocking wear, h/day | 0 | 0.0% |
| Prior lower-extremity or pelvic surgery | 0 | 0.0% |
| Regular exercise | 0 | 0.0% |

*No missing values were reported for the variables in the supplied revised dataset.*

**Table S2. Detailed bootstrap internal validation of the Cox prediction model**

| **Measure** | **Apparent** | **Corrected** | **Optimism** |
| --- | --- | --- | --- |
| Somers' Dxy | 0.4534 | 0.3810 | 0.0724 |
| R² | 0.2196 | 0.1513 | 0.0683 |
| Calibration Slope | 1.0000 | 0.8072 | 0.1928 |
| D | 0.0590 | 0.0368 | 0.0222 |
| U | -0.0027 | 0.0079 | -0.0106 |
| Q | 0.0617 | 0.0289 | 0.0328 |
| g | 0.7391 | 0.5612 | 0.1779 |

*The corrected calibration slope was 0.8072 in this detailed bootstrap output.*

**Table S3. Sensitivity analyses of the multivariable Cox model**

| **Variable** | **Primary HR (95% CI)** | **P value** | **Exclude time 0 HR (95% CI)** | **P value** | **Clinical-class-adjusted HR (95% CI)** | **P value** |
| --- | --- | --- | --- | --- | --- | --- |
| Age (per year) | 1.024 (0.999-1.050) | 0.064 | 1.022 (0.996-1.048) | 0.097 | 1.016 (0.989-1.044) | 0.240 |
| Male (vs Female) | 0.936 (0.576-1.521) | 0.788 | 0.922 (0.566-1.501) | 0.743 | 0.991 (0.601-1.633) | 0.971 |
| BMI (per kg/m²) | 1.122 (1.013-1.242) | 0.027 | 1.106 (0.997-1.227) | 0.056 | 1.136 (1.028-1.256) | 0.013 |
| Disease duration (per year) | 1.024 (1.000-1.048) | 0.046 | 1.026 (1.002-1.050) | 0.032 | 1.029 (1.002-1.056) | 0.036 |
| Family history (Yes vs No) | 1.635 (1.017-2.628) | 0.042 | 1.681 (1.044-2.708) | 0.033 | 1.591 (0.968-2.615) | 0.067 |
| Perforator (Yes vs No) | 0.351 (0.193-0.639) | <0.001 | 0.333 (0.182-0.608) | <0.001 | 0.281 (0.152-0.521) | <0.001 |
| GSV diameter (per mm) | 1.022 (0.926-1.128) | 0.666 | 1.013 (0.917-1.119) | 0.793 | 1.032 (0.931-1.143) | 0.550 |
| VCSS (per point) | 1.040 (0.977-1.107) | 0.223 | 1.038 (0.974-1.105) | 0.254 |  |  |

*The clinical-class-adjusted model did not report VCSS in the supplied sensitivity-analysis file.*

**Table S4. Variance inflation factors for primary model predictors**

| **Variable** | **VIF** |
| --- | --- |
| Sex | 1.073 |
| BMI, kg/m² | 1.046 |
| Disease duration, years | 1.028 |
| Family history of varicose veins | 1.023 |
| Perforator status | 1.076 |
| GSV diameter, mm | 1.027 |
| VCSS, points | 1.008 |

*All VIF values were close to 1, indicating little evidence of multicollinearity.*

**Table S5. Outlier-screening summary**

| **Variable** | **Flagged outlier count** |
| --- | --- |
| Age, years | 0 |
| BMI, kg/m² | 1 |
| Disease duration, years | 3 |
| GSV diameter, mm | 0 |
| VCSS, points | 2 |
| Compression stocking use, months | 5 |
| Daily compression stocking wear, h/day | 11 |
| Follow Time | 0 |

**Table S6. Compact model-validation summary**

| **Metric** | **Value** |
| --- | --- |
| Original C-index | 0.7267 |
| Bootstrap-corrected C-index | 0.6935 |
| Calibration slope | 0.8212 |

*The calibration slope reported in this compact summary (0.8212) differs slightly from the corrected slope in the detailed bootstrap output (0.8072; Table S2). Both values are retained exactly as supplied.*

**Table S7. Relative variable importance in the multivariable Cox model**

| **Variable** | **Wald χ²** | **Relative importance** |
| --- | --- | --- |
| Perforator | 11.742 | 39.27% |
| BMI | 4.879 | 16.32% |
| Family history | 4.119 | 13.77% |
| Disease duration | 3.987 | 13.33% |
| Age | 3.434 | 11.48% |
| VCSS | 1.482 | 4.96% |
| GSV diameter | 0.187 | 0.62% |
| Sex | 0.072 | 0.24% |

**Supplementary Figures**

*Figure S1. Kaplan-Meier recurrence-free survival curves. (A) Family history (log-rank P=0.033). (B) Perforator vein incompetence (Yes = present; No = absent; log-rank P<0.0001).*

*Figure S2. Forest plot of the primary multivariable Cox regression. Point estimates are hazard ratios with 95% confidence intervals; the dashed vertical line indicates HR=1. “Perforator” denotes perforator vein incompetence (Yes = present; No = absent).*

*Figure S3. Exploratory restricted cubic spline displays for BMI and disease duration in the multivariable Cox model. Shaded regions represent uncertainty bands. No formal nonlinearity P values were supplied.*

*Figure S4. Relative variable importance in the multivariable Cox model based on Wald χ² contribution.“Perforator” denotes perforator vein incompetence.*

*Figure S5. Pairwise correlation matrix for age, BMI, disease duration, GSV diameter, and VCSS. The largest displayed correlation is age with disease duration (r=0.52).*
